# Systematic literature review and meta-analysis of the relationship between adherence, competence and outcome in psychotherapy for children and adolescents

**DOI:** 10.1007/s00787-018-1265-2

**Published:** 2019-01-02

**Authors:** Hannah Collyer, Ivan Eisler, Matt Woolgar

**Affiliations:** Institute of Psychiatry, Psychology and Neuroscience, King’s College London, London, UK

**Keywords:** Adherence, Competence, Fidelity, Therapy, Child, Adolescent

## Abstract

The extent to which therapist adherence to guidelines and clinician skill or competence may play a role in the prediction of therapeutic outcomes remains inconclusive. This systematic literature review and meta-analysis considers whether adherence or competence predicts youth outcome in child and adolescent psychotherapy, and whether there are any identifiable factors which moderate the strength or direction of this relationship. A systematic literature search identified 35 studies in 52 papers. The studies contained 29 effect sizes for the relationship between adherence and outcome, while nine effect sizes were extracted for competence, and a further five effects measured a composite of adherence and competence constructs, referred to as fidelity in this report. The meta-analysis indicated a small but significant relationship between therapist adherence and outcome, although the small size of effect suggests that outcomes are likely to be more strongly associated with factors other than adherence. No significant relationship was identified between competence or composite fidelity and outcome. Although variance was observed in effect sizes, no significant moderation by client group, intervention type, or implementation measure informant was identified. Further study is needed to understand the specific circumstances under which adherence and outcome are related.

**Electronic supplementary material:**

The online version of this article (10.1007/s00787-018-1265-2) contains supplementary material, which is available to authorized users.

## Introduction

Treatment fidelity is often used to refer to a range of processes involved in model delivery. The classification of this construct varies across studies, often including characteristics such as dosage and differentiation [1], as well as the two processes which are the focus of the present investigation, therapist adherence and competence. Therapist adherence refers to the accuracy with which the specified elements of an intervention model are implemented, while competence refers to how skilfully the intervention is delivered [2].

Proponents of manualised models or empirically supported treatments (ESTs) argue that variability in outcomes when transporting interventions from research settings could be attributed to variable therapist adherence or competence, rather than failures in the therapeutic model. This suggests that the key to transporting findings from well-controlled efficacy studies to everyday clinical settings is to ensure high levels of therapist adherence and competence in the delivery of the therapeutic model [3].

Critics, on the other hand [4], have argued that the assumptions of manualised interventions are not always well substantiated. For example, adherence is not consistently seen to be associated with outcome, and trials often fail to test the effectiveness of individual components within the therapy. It has also been argued that operationalizing the manual as being the treatment itself (at least implicitly but often explicitly) significantly reduces the fundamental role of clinical judgement [5]. Understanding the role of therapist adherence and competence in predicting treatment outcomes has important implications for our understanding of evidence-based therapeutic practice.

Characteristics of the therapeutic context can influence the therapist’s ability to deliver the intervention with adherence and competence. Factors which might influence practice include client complexity or motivation, or the level of training and supervision provided [6–9].

There is some indication that the relationship between therapist adherence or competence and outcome is variable across intervention types and client groups. For example, there is considerable evidence that therapist adherence plays a role in predicting outcomes in multisystemic therapy (MST) for adolescent antisocial behaviour [10], but there is less consistent evidence of such a relationship in cognitive-behavioural interventions [3]. Further, adolescent substance use outcome has been associated with therapist competence in motivational interviewing (MI) [11], but was not found to be associated with competence in cognitive behavioural therapy and multidimensional family therapy (MDFT) [12]. These findings suggest that adherence and competence may play a more prominent role in some interventions than others.

However, even within the same intervention type, the relationship between implementation and outcome can be variable. This may relate to methodological differences across studies. Variation in approaches to measurement is seen across studies, which may affect the validity of ratings. For example, adherence rated by independent observers may be less subject to bias or demand characteristics than supervisor, therapist or client ratings [13]. Inconsistencies between studies may also be due to moderation by other variables. For instance, Barber et al. [14] found that therapist adherence was less strongly related to outcome when therapeutic alliance was strong. Similarly there is evidence that in the context of a poor therapeutic alliance, strong adherence is associated with worse outcome [15]. Further, Huppert et al. [9] found that therapist adherence predicted poorer outcome in clients with low motivation, but was not related to outcome in clients with high motivation. However, the role of moderators has not been extensively studied and, therefore, understanding of the characteristics which might affect the relationship between implementation and outcome remains limited.

Some research has also indicated a curvilinear relationship between therapist adherence and outcome [14]. This would suggest that rigidly high adherence may limit the opportunity for the therapist to respond to individual needs, while low adherence may lead the therapist to miss out many key elements of the therapy. Barber et al. [14] also propose a potential interaction between therapist adherence and competence, suggesting that where competence is high, moderate rather than high adherence may lead to the best outcomes. Although no such relationship was found, these authors suggest that overall high levels of competence reduced the chance of detecting this effect and that further study is warranted.

Syntheses of the evidence have attempted to make sense of the contradictory findings across the evidence base. A meta-analysis of individual psychotherapy, comprising mainly adult intervention studies, found that when controlling for therapeutic alliance, neither adherence nor competence predicted outcome particularly well, with the exception of a potential link between competence and outcome for certain interventions such as those for major depression [16]. These findings suggest that there is limited association between implementation and outcome for individual and primarily adult intervention. In contrast, a systematic review between adherence and outcome in child and adolescent mental health indicated that there was an association [17]. This suggests a more consistent relationship between implementation and outcome in child and adolescent intervention than with adults.

However, syntheses focussing on specific intervention approaches or client groups within child and adolescent psychotherapy show variation in the association between implementation and outcome. A recent systematic review of child and adolescent cognitive behavioural therapy concluded that the understanding of the relationships between adherence or competence and outcome remains inconclusive [18]. In contrast, a recent meta-analysis has indicated a relationship between integrity and outcome in intervention for juvenile antisocial behaviour [19]. These findings suggest, in line with inferences drawn based on individual studies, that implementation may be associated with outcome in some child and adolescent intervention approaches but not others.

Much of the evidence synthesis so far for child and adolescent psychotherapy relies on qualitative review or a narrow focus on specific intervention approaches or client groups. In light of this, the present meta-analysis aims to provide an updated, comprehensive quantitative synthesis across this complex evidence base. The primary research question aims to understand whether youth outcome in child and adolescent psychotherapy is predicted by (a) adherence, (b) competence and (c) composite fidelity. Composite fidelity can be variably defined in the literature, but for the purpose of this study is used to refer to a composite of adherence and competence.

A secondary research question considers characteristics which previous research has indicated may act as potential moderators of the strength of the relationship between implementation and outcome but which are not yet fully understood. This will test whether the size of any observed association between implementation and outcome is moderated by (a) intervention modality (i.e. the nature of the therapeutic approach) (b) clinical group (i.e. the diagnostic characteristics of the focus client group) or (c) adherence or competence informant (i.e. whether implementation is rated by clients, therapist, supervisor or an independent observer).

## Method

This review was prospectively registered on PROSPERO International Prospective Register of Systematic Reviews (ID Number CRD42016046671). Procedures and reporting are in accordance with the recommendations of the PRISMA guidelines [20, 21].

### Literature searches

Systematic literature searches were completed up to 21st July 2018 using PsychINFO, Embase and Medline databases. Searches and data extraction were carried out by the lead author.

### Search terms

- Fields: Abstract, Keyword, Subject Headings, Title.
- Terms: (Fidelity or adherence or integrity or competence or implementation) and (treatment or therapy or psychotherapy or intervention or therapist or therapeutic or counselling) and (child or adolescent or parent or parenting or teenager or teenage or children or adolescents or youth).

Full details of search and exclusion terms are available in Online Resource 1. Duplicates were removed from the search results and the title and abstract of each article were screened, followed by screening of all remaining full text articles. Relevant articles referenced in the obtained studies were also considered for inclusion.

### Inclusion and exclusion criteria

Included articles were those reporting a primary study of an outpatient psychosocial targeted intervention for mental health or behaviour, within a clinical population of children and adolescents up to age 21. Studies were included which reported statistical analysis of the relationship between a quantifiable measure of either therapist adherence, competence or composite fidelity at the therapist or client level and a measure of youth mental health or behavioural outcome. Interventions delivered by teachers, unqualified school professionals, caregivers or peers were excluded, as were universal interventions or those targeting health or educational outcomes. All included studies were reported in English language and published in a peer review journal.

### Study quality

Included studies were critically appraised for study quality by the lead author, using criteria to assess power and risk of bias. Given that there are no consistently established guidelines for assessing study quality in non-experimental studies such as the observational and correlational designs included in the present analysis, the included studies were assessed according to eight pre-defined binary criteria, established based on recommendations from a number of similar previous reviews [22–24]. Studies which met the following criteria were rated as being of higher quality:

- Reported power ≥ 80%, or sample size ≥ 53 per group [25, 26].
- Reported participation rate ≥ 70% of eligible or approached sample [23].
- Dropout at follow-up ≤ 30%, or missing data shown to not differ from those with complete data on any of the predictor variables, or showing that predictor–outcome relationships remained the same after adjusting for missing data [23].
- Used a reliable diagnostic measure or rating scale, or clear selection criteria reported [24].
- External or independent observer rated measure of adherence or competence, measured across multiple time points (unless a one-session intervention), and inter-rater agreement established in the study or use of coders trained to this level according to the following criteria: intraclass correlation coefficient (ICC) ≥ 0.60 [27] or Kappa ≥ 0.61 [28], or percent agreement ≥ 90% [29], or the score is based on agreement by multiple raters.
- Used questionnaire outcome measures which demonstrate reliability and validity in the present or previous studies (internal consistency: *α* ≥ 0.70 [22], convergent validity: *r* = 0.6 [30] or interrater reliability: ICC ≥ 0.60 [27] or Kappa ≥ 0.61 [28]) or other type of outcome measure with low risk of bias (e.g., objective measure such as urine screening).
- Controlled or adjusted for the influence of baseline symptom severity, or used a measure of change.
- Reported adjustment or control for influence of potential confounders, or indication that potential confounders do not relate to outcome.

### Meta-analysis

#### Data extraction and preparation

Effect sizes for included studies were classified according to the constructs of adherence and competence as defined in the introduction, while the term fidelity was used to classify studies reporting a composite of these two constructs. Where papers reported on the same or overlapping samples, a single effect size was used to ensure that each meta-analysis included only one independent effect size from each subject sample. The effect size selected for inclusion was that reported at the earliest outcome timepoint, or where different sample sizes were used, the largest sample. Where a range of outcomes were measured, effects were included only for those addressing the primary problem in each study (see Online Resource 2). To avoid inclusion of statistically dependent effect sizes, which can threaten validity [31], an average effect size was calculated where multiple primary outcomes were measured, where separate effect sizes were reported for multiple informants on the same measure, or where effects were reported for multiple adherence or competence components. Where multiple follow-up time points were reported, the earliest post intervention time point for which sufficient data were available was selected, most often a measurement directly post intervention.

The selected effect size, Pearson’s correlation coefficient (*r*), was directly reported in several studies. Results were standardised such that positive correlations were given to findings in the direction of our prediction, i.e. increased adherence or competence predicted therapeutic improvement. Where *r* was not directly reported, conversions were made from other effect sizes or from reported test statistics [32–34]. When sufficient statistical data were not available but effects were reported to be non-significant, the *r* value was estimated conservatively as zero. Remaining studies reporting significant effects but with insufficient data to compute effect sizes were excluded from the analysis.

Each study was coded for three multi-level categorical moderators: clinical diagnostic group [emotional disorders, child and adolescent behaviour problems, substance use, autism spectrum disorder (ASD)], intervention modality [cognitive behavioural therapy (CBT), family therapy, parenting, other youth non-CBT intervention (individual or group), other non-CBT intervention with youth and parent components (individual or group)] and adherence or competence informant (observer, therapist, client report, supervisor report, composite of client and therapist ratings).

### Statistical analysis

Analysis was completed using SPSS version 25 [35] using syntax from Lipsey and Wilson [36] based on Hedges and Vevea [37] random effects meta-analysis. Estimated mean population effect and its significance was computed as an average of standardised Fisher’s *z* transformed effect sizes, weighted by inverse variance as a function of sample size (*n* − 3). A test of homogeneity established whether any variance in effect sizes is likely due to sampling error alone or systematic differences between studies. For significant effects, a sensitivity analysis tested how conclusions might be affected if studies at higher risk of bias were excluded from the analysis. Excluded studies were those scoring low quality in four or more of the eight areas against which they were rated.

A model analogous to analysis of variance examined whether moderator characteristics are associated with heterogeneity of effect sizes, by testing whether overall weighted effect size differs significantly between categories of each moderator. Moderator levels with only one available effect size could not be included in the moderation analysis.

Risk of publication bias was assessed using a rank correlation test of the strength or significance of the relationship between standardised effect size and associated variance [38]. The potential impact of any publication bias was estimated using a sensitivity analysis using syntax for SPSS and R [39] which computed adjusted estimates of effect size based on models estimating the impact of moderate or severe bias [40].

## Results

**Fig. 1:**
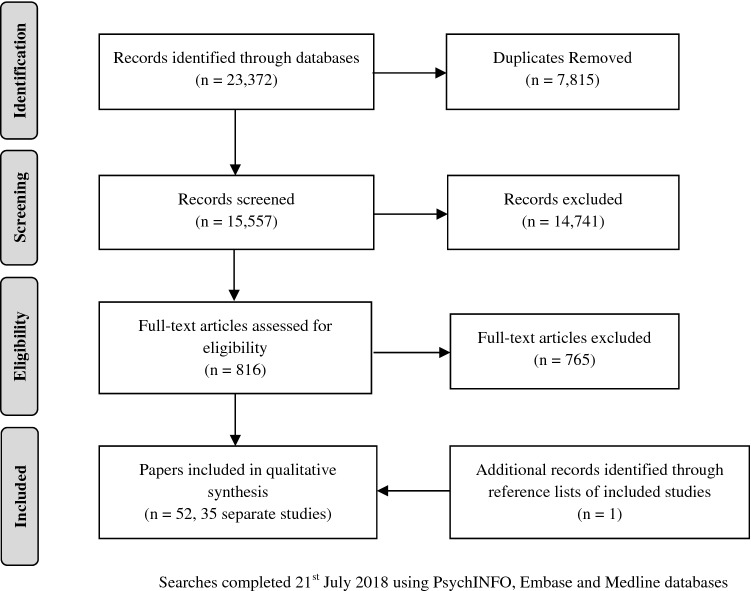
Prisma flow chart of study identification

The search returned 23,372 papers. 7815 duplicates were removed, 14,741 were excluded based on title and abstract and a further 765 were excluded based on fulltext (see Fig. 1). The remaining 51 papers met inclusion criteria, and one additional paper [41] was identified as a reference within one of the included studies. The final review, therefore, included 52 papers covering 35 separate studies. The complete list of included papers and their characteristics is presented in Table 1, with further detail available in Online Resource 2.

**Table 1:** Details of included Studies

Study	Client group	Intervention	Sample Size	Age range (mean, SD)	Primary outcome measure(s) used in meta-analysis
[50]	Families in crisis	Family crisis intervention program (FCIP)	183	0–18 (11.54, 5.17)	Parent and youth rated Strengths and Difficulties Questionnaire (SDQ)
[51]	Disruptive/withdrawn	Early risers “skills for success” program	262	Grade 1 and 2 (6.92, 0.93)	Teacher rated Behavioural Assessment System for Children-II (BASC-2)
[80]	Attention-deficit/hyperactivity disorder (ADHD)	Cognitive behavioural therapy (CBT) (solution-focused treatment)	36	12–17 (14.28, 1.19)	Dutch BRIEF (inventory of executive function) plan/organise scale
[52]	Behavioural problems	Family brief and intensive intervention (BII)	160	6–17 (14.7, 2.1)	Placement/services required
[71]^a^	Conduct Disorder	Incredible years parent group	86	36–59 months (46.1 months, 6)^A^	Parent rated Eyberg Child Behaviour Inventory (ECBI) intensity and problem scales
[81]	Risk of depressive disorder	Youth single session brief motivational interviewing (MI)	43	14–21 (17.44, 2.17)	Youth CES-D-10 (depression scale)
[43]	Substance abuse	Youth adolescent community reinforcement approach (A-CRA) (and parent and family sessions)	399	12–18 (N/A)	Alcohol and other drug use
[53]^b^	Substance abuse	Youth A-CRA (an parent and family sessions)	953	N/A (15.8, 1.4)	Global appraisal of individual needs (GAIN)—substance use remission status
[54]	Substance using youth offenders	Multisystemic therapy (MST)	40	12–17	Arrest record; self-report alcohol and cannabis use; Self-Report Delinquency scale (SRDS)
[55]	Depression symptoms	Youth group CBT	147	11–12 (N/A)	Youth report Clinical Depression Inventory (CDI)
[46]^c^	Anxiety disorder	Individual CBT (and parent sessions)	17	7–17 (11.12, 2.75)	Clinician anxiety disorders interview schedule (ADIS-C/P); parent and youth rated screen for child anxiety—related emotional disorders (SCARED)
[56]	Behavioural problems	Functional family therapy (FFT)	98	N/A (14.05, 1.93)	Parent and youth rated SDQ
[57]	Behavioural problems	FFT	42	N/A (14.22, 1.45)	Parent rated SDQ
[84]	Incarcerated youth with antisocial behaviour	Youth group CBT	89	N/A (15.54, 1.56)	Youth rated social skills; moral value and moral Judgement; cognitive distortion
[10]^d^	Violent and chronic youth offenders	MST	82	10–17 (15.22, N/A)^A^	Arrests and incarceration; SRDS; Parent rated Problem Behaviour Checklist (RPBC)
[58]	Substance using youth offenders	MST	58	12–17 (15.7, 1.0)^A^	Self-report alcohol/marijuana
[59]	Behavioural problems	FFT	59	9–16 (13 years 7 months)	Composite measure of parent-rated conduct problems, parent- and youth-rated alcohol and substance use frequency, and SRDS
[12]^e^	Substance use and behaviour problems	Family multidimensional family therapy (MDFT) and youth CBT	74 MDFT, 62 CBT	13–17 (15.5, 1.3)	Time line follow-back (TLFB) cannabis frequency
[60]	Substance use disorder	MST	41	13–17 (15 years 10 months, 14 months)	Self-report cannabis abstinence
[82]^f^	Externalising behaviour	Parent management training (individual)	331	04–12 (8.7, N/A)	Parent child behaviour checklist (CBCL) and parent daily report (PDR); teacher report form (TRF)
[78]^g^	Antisocial/problem behaviour	MST	4290	12–17 (15.62, 1.38)	Living at home; engaged in school or work; no new arrests
[47]	Anxiety disorder	Individual and group CBT (and parent sessions)	52	08–12 (10.22, 1.15)	Clinician ADIS-C/P; Youth MASC (anxiety scale); parent CBCL internalising scale
[63]^h^	Behaviour/conduct disorder	MST	973	12–17 (N/A)	Criminal behaviour at completion of treatment
[85]	Externalising behaviour	Parent Management Training Oregon (PMTO)	46	4–12 (7.85, 2.36)	Parent-rated CBCL and teacher-rated TRF (Dutch versions)
[11]	Cannabis use	Youth single session MI	75	16–19 (18, N/A)	Cannabis cessation
[62]	Exposure to interparental violence	Youth and parent trauma-focused psycho-education	100	06–12 (9.35, 1.55)	Parent report TSCYC; youth report TSCC (trauma symptom checklists)
[64]	Anxiety disorder	Youth individual CBT (and parent sessions)	279	07–17 (10.76, 2.79)	Clinician PARS (anxiety scale); youth MASC (anxiety scale); parent-rated CBCL anxiety/depression scale
[42]	Drug abuse	Brief strategic family therapy (BSFT)	246	12–17 (15.5, 1.3)	TLFB substance use
[8]	Cannabis use disorder	MDFT	Europe (212) US (171)	13–18 (Europe: 16.3, 1.2)^A^ (US: 15.6, 1.1)	TLFB substance use frequency and problem
[44]^i^	Serious antisocial behaviour	MST	1979	N/A (14.0, 2.35)	Parent rated CBCL externalising
[49]	Youth offenders	FFT	431	13–17 (N/A)	Recidivism
[45]	Social, emotional, and behavioural problems	Youth supportive-expressive group counselling	266	10–18 (N/A)	Self-report aggression
[83]^j^	Elevated problem behaviour	Family check-up	79	2 years 0 months-2 years 11 months (29.9 months, 3.2)	Parent-rated CBCL externalising
[65]^k^	Autism spectrum disorder	Youth and parent early intensive behavioural intervention (EIBI)	24	26-81 months (55.67 months, 17.63)	Observer-rated child challenging behaviour
[72]	Serious emotional disturbance	Youth and parent child psychiatric rehabilitation (CPSR)	79	N/A (10.31, 3.54)	Parent-rated youth counselling impact scale (YCIS)

^A^Age is reported for whole sample including control

Studies above were also reported in the following identified papers:^a^[73], ^b^[74], ^c^[75], ^d^[76, 77], ^e^[41, 67], ^f^[86], ^g^[78], ^h^[66], ^i^[7, 48, 68–70], ^j^[87], ^k^[79]

### Characteristics of identified studies

The client groups in the included studies were children and adolescents with behaviour problems including offending (*N* = 18, 51%), substance abuse (*N* = 9, 26%), emotional disorders (*N* = 7, 20%) or autism spectrum disorder (ASD) (*N* = 1, 3%). Interventions were primarily family therapy (*N* = 16, 46%), or cognitive behavioural therapy (CBT) (*N* = 7, 20%), as well as parenting (*N* = 4, 11%), youth non-CBT intervention (*N* = 3, 9%) and non-CBT intervention with youth and parent components (*N* = 6, 17%). Detail on the specific interventions within each category is available in Online Resource 3.

Measures of therapist adherence, competence and composite fidelity differed in content and complexity. Some studies measured the frequency of implementation of certain core strategies [42], whilst others also incorporated an evaluation of thoroughness of their use [8]. There was also variation in the number and timing of assessments from which overall scores were based such that some interventions measured implementation measures at every session [43], some at regular intervals [44], or specific pre-planned sessions [45], whilst others took ratings from one or more randomly selected sessions over the course of therapy [46, 47].

The relationship between adherence and outcome was measured in 29 studies across 43 papers [7, 8, 10–12, 41–78]. Adherence was rated most frequently by observers (*N* = 13, 45%), as well as therapists (*N* = 4, 14%), clients (*N* = 4, 14%), supervisors (*N* = 4, 14%), or a composite of client and therapist ratings was used (*N* = 4, 14%). A significant relationship between adherence and at least one youth outcome was reported in 24 studies (83%), while five (17%) reported no relationship.

The relationship between competence and outcome was measured in nine studies across ten papers [11, 12, 46, 51, 53, 59, 64, 74, 79, 80]. Competence was rated mostly by observers (*N* = 7, 77%), and in two instances by supervisors. A significant relationship between competence and at least one youth outcome was reported in five studies (56%), while four (44%) found no relationship.

The relationship between composite fidelity and outcome was measured in five studies across seven papers [81–87]. Composite fidelity was rated mostly by observers (*N* = 4, 80%), and in one instance by supervisors. A significant relationship between composite fidelity and at least one youth outcome was reported in two studies (40%), while three (60%) found no relationship.

A small number of studies considered potential moderators of the strength or direction of the relationship. Sexton and Turner [49] found an interaction for youth risk, such that adherence more strongly predicted outcome in the presence of high peer risk. Schoenwald et al. [44] found an interaction between therapist adherence and organisational structure and climate, such that therapist job satisfaction predicted improvements in behaviour only when therapist adherence was low. Two studies measuring alliance found no interaction with the relationship between therapist adherence or fidelity and outcome [47, 82], whilst one study found the relationship between therapist competence and outcome became non-significant when controlling for alliance [80]. In the two studies in which it was investigated, an interaction between adherence and competence was not found in the prediction of outcomes [11, 12].

### Study quality

Complete details of study quality ratings are available in Online Resource 4. 43% of studies (*N* = 15) reported a low rate of uptake of the intervention or research study by eligible cases, and 40% of studies (*N* = 14) reported risk of bias due to dropout or missing data from the study. 46% of studies (*N* = 16) also failed to report clear and valid selection criteria. Finally, low power or small sample size was identified in 34% of cases (*N* = 12).

Risk of bias in the reliability and validity of the adherence or competence measures was identified in 66% of studies (*N* = 23). For most studies, this risk of bias related to using a non-independent informant (i.e. therapist, client or supervisor) rather than an observer (17 studies). Where observer ratings were used, and inter-rater reliability was reported, the majority of studies met the inter-rater reliability thresholds, indicating that the reliability of observer ratings was rarely a concern. Inter-rater reliability scores for each study are presented in Online Resource 2. However, only 11% of studies (*N* = 4) reported outcome measures at risk of bias. Those that did use subjective ratings such as therapist reported criminal behaviour [63] or adapted forms of validated measures [84], rather than validated questionnaires.

Baseline symptom severity was controlled for in 83% of studies (*N* = 29), such that any relationship between implementation and outcome was independent of the influence baseline severity may have on both factors. However, 43% of studies (*N* = 15) failed to report consideration of any other confounding variables, and amongst those which did so there was considerable variation in controlled variables. These included demographic or treatment variables such as age or gender (*N* = 8), dosage, time in treatment or assessment interval (*N* = 5), parent marital status (*N* = 4), income (*N* = 3) and therapeutic alliance (*N* = 3).

### Meta-analysis

Two adherence studies and one competence study with insufficient data to compute effect sizes were excluded from the analysis [71, 72, 79]. Two papers reported outcomes for two independent samples [8, 12].

**Fig. 2:**
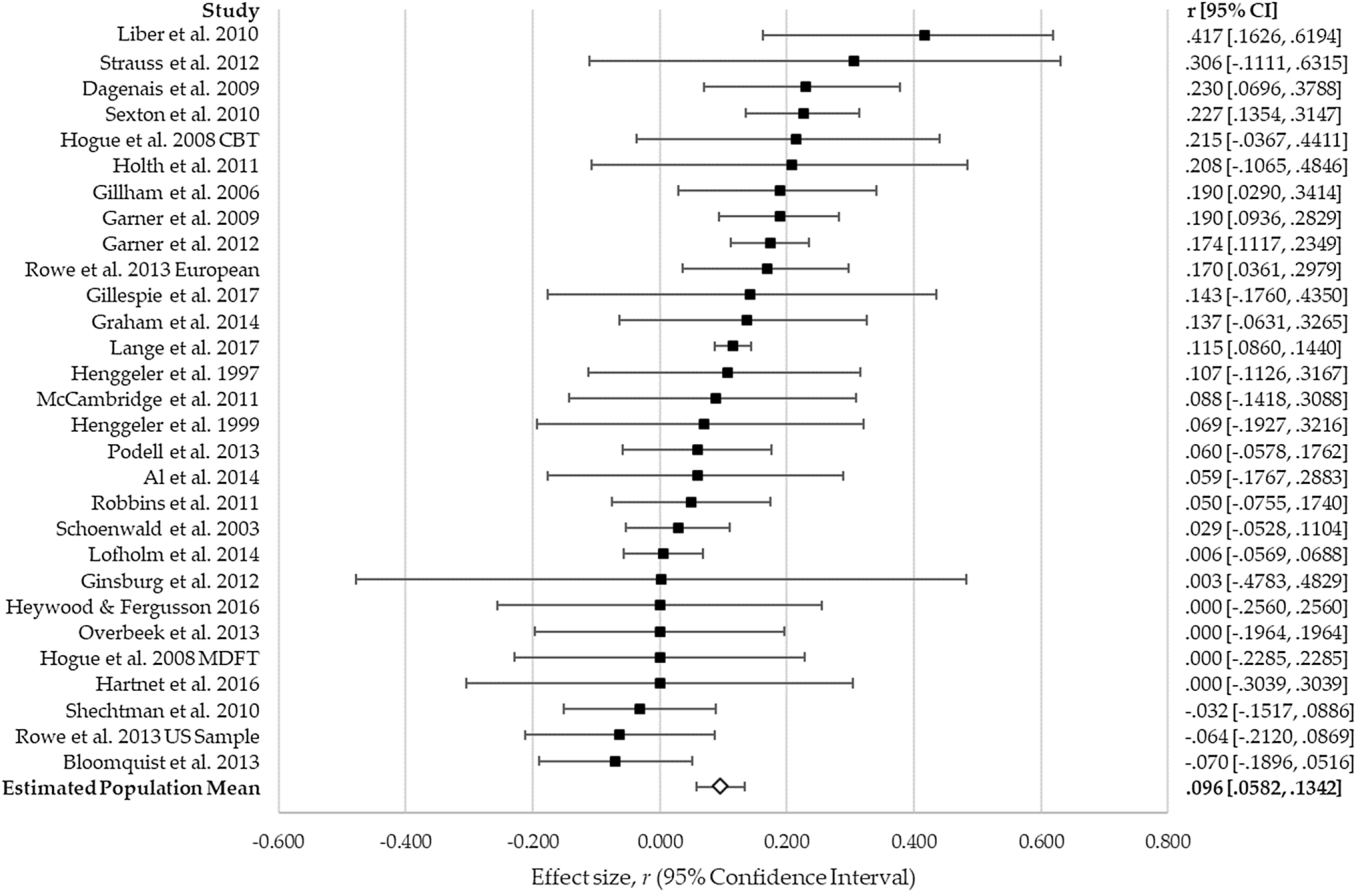
Forest plot of adherence–outcome effect sizes

The 29 adherence-outcome effect sizes ranged from − 0.070 to 0.444 (see Fig. 2), and a small but statistically significant relationship between therapist adherence and outcome was identified, *r* = 0.096 (95% CI = 0.058, 0.134), *z* = 4.938, *p* < 0.001 (see Table 2). Variance in effect sizes was significantly greater than would be expected by sampling error alone [*Q* (28) = 62.352, *p* < 0.001] and, therefore, likely affected by differences between studies, although no significant moderation effect was identified (see Table 3). However, consideration of individual effects for each moderator group suggested a small number of circumstances under which adherence was not significantly associated with outcome. These were youth non-CBT intervention (*r *= 0.006, 95% CI = − 0.145, 0.158, *z* = 0.082, *p *=* 0*.935), and where client informants were used to rate adherence (client informant *r *= 0.040, 95% CI = − 0.034, 0.113, *z* = 1.049, *p *= 0.294; client and therapist composite: *r *= 0.119, 95% CI = − 0.048, 0.280, *z *= 1.400, *p *= 0.162). All other moderator categories tested were significantly associated with outcome (clinical group categories: *r* = 0.071–0.127; intervention type categories: *r *= 0.089–0.169; informant categories: *r *= 0.105–0.148).

**Table 2:** Meta-Analysis

	Distribution of effect size			Estimated population mean					Test of homogeneity		
	*N*	Min	Max	*r*	95% CI		*Z*	Sig.	*Q*	*df*	Sig.
Adherence	29	− 0.070	0.444	0.0964	0.0582	0.1342	4.9384	0.000	62.3516	28	0.000
Competence	9	0.000	0.173	0.0264	− 0.0199	0.0726	1.1188	0.263	2.5951	8	0.957
Composite fidelity	5	− 0.273	0.213	0.0615	− 0.0702	0.1911	0.9153	0.360	7.6997	4	0.103

**Table 3:** Moderation analysis

	Clinical group			Intervention type			Informant		
	*Q*	*df*	Sig.	*Q*	*df*	Sig.	*Q*	*df*	Sig.
Adherence	1.4580	2	0.482	3.0602	3	0.382	3.5283	4	0.474
Competence	1.3663	2	0.505	0.7467	2	0.688	–	–	–
Composite fidelity	–	–	–	–	–	–	–	–	–

Moderator levels with only one available effect size could not be included in the moderation analysis

**Fig. 3:**
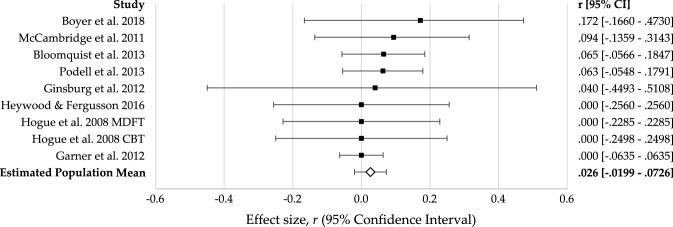
Forest plot of competence–outcome effect sizes

The nine competence-outcome effect sizes ranged from 0.000 to 0.173 (see Fig. 3); however, competence did not have a statistically significant association with outcome, *r *= 0.026 (95% CI = − 0.020, 0.073), *z* = 1.119, *p* = 0.263 (see Table 2). There was no significant variance in effect sizes [*Q* (7) = 2.595, *p* = 0.957] indicating the studies likely represent a common population mean. Although there were insufficient levels to test informant as a moderator, there was no significant moderation effect for clinical group or intervention modality (see Table 3).

**Fig. 4:**
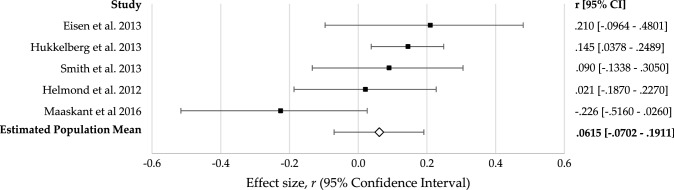
Forest plot of composite fidelity–outcome effect sizes

The five fidelity-outcome effect sizes ranged from − 0.273 to 0.213 (see Fig. 4); however, composite fidelity did not have a significant association with outcome, *r *= 0.06 (95% CI = − 0.070, 0.191), *z* = 0.9153, *p* = 0.360 (see Table 2). There was no significant variance in effect sizes (*Q* (4) = 7.700, *p *= 0.103), indicating the studies likely represent a common population mean, although there were insufficient levels to test any moderation analysis for this effect.

### Sensitivity analysis

Using a subsample of 22 adherence effect sizes with the lowest risk of methodological bias, the overall size of effect remained very similar to the effect seen in the main analysis when all effects were included (*r* = 0.097 (95% CI = 0.052, 0.141), *z* = 4.253, *p* < 0.001). Tests for heterogeneity also indicated that variance in effect sizes remained significantly greater than would be expected by sampling error alone [*Q* (21) = 44.579, *p* < 0.01], although the extent of observed variation was reduced.

### Publication bias

The Begg and Mazundar [38] random effects rank correlation test (see Table 4) indicated no risk of publication bias, although the test may have lacked power to detect an effect with the relatively small sample size. Sensitivity analysis indicates that correction for moderate publication bias would reduce the strength for all effects, and correction for severe publication bias could reverse the direction (see Table 4).

**Table 4:** Publication bias

	Random effects rank correlation test		Sensitivity analysis		
			Unadjusted estimate (*r*)	Adjusted estimate (*r*) One-tailed	
	*τ*	Sig.		Moderate	Severe
Adherence	0.084	0.524	0.0966	0.0723	− 0.1922
Competence	0.167	0.532	0.0265	0.0146	− 0.0011
Composite fidelity	− 0.200	0.624	0.0829	0.0511	− 0.3553

## Discussion

Three separate meta-analyses tested the relationship between therapist adherence, competence or composite fidelity and youth outcome in child and adolescent psychotherapy, and whether this relationship was moderated by client group, intervention modality or implementation measure informant.

A small but statistically significant relationship was identified between therapist adherence and youth outcome, suggesting that implementing the appropriate components of therapy plays a small role in child and adolescent therapeutic practice. This finding is in line with a previous qualitative review of child and adolescent mental health intervention and prevention [17], and with a meta-analysis of intervention for juvenile antisocial behaviour [19], although it appears inconsistent with the meta-analysis by Webb et al. [16]. This suggests, if taken at face value, that therapist adherence may be marginally more influential in child and family settings compared with adult individual therapy settings. However, the finding of a statistically significant association with outcome is tempered by the fact that the small effect suggests that adherence only accounts for just under one percent of variance in outcomes.

In line with the meta-analysis by Webb et al. [16], youth outcome was not significantly related to therapist competence, perhaps not surprising given the limited role that adherence was found to have. This of course does not mean that clinical competence is unrelated to outcome but simply that competence in implementing model specific skills may be less important than more general clinical skills. However, the non-significant findings may also result from limited variance in therapist competence such that competence is not often rated as low. Furthermore, the role of competence is not widely measured or reported across studies at present, limiting the extent to which this effect has been tested across settings. The present analysis also identified no relationship found between composite fidelity and outcome. This appears to be in line with the finding that therapist adherence and competence are differentially related to outcome, suggesting that it may be more informative to measure these as separate constructs. The findings relating to competence and fidelity, however, need to be viewed with considerable caution given the small number of studies in these analyses.

Although significant variance was detected in adherence effects, effect sizes were not moderated by intervention modality, clinical diagnostic group or adherence informant. This finding may suggest that the small but significant association between therapist adherence and outcome is present across diverse intervention types and client groups and is stable across adherence informants. The moderator categories where the adherence–outcome relationship was not apparent (i.e. youth non-CBT intervention, and client informant) may be an artefact of the small numbers of effect sizes within these subgroups and, therefore, reduced power to detect an effect. However, given observed variance in effects indicates that the mean effect size does not represent a single population, and the absence of moderator effects suggests that there may be other unmeasured or undetected explanations for the variance in adherence effects. Unmeasured moderators may include the time lag between the measurement of implementation and outcome, where a shorter time lag might be expected to be associated with a larger effect. However, given heterogeneity in measurement approaches observed, developing a consistent variable measuring this was out of scope for the current study. Other potential moderators of the relationship between adherence and outcome not measured in the present analysis may include client characteristics, such as likelihood to respond to intervention [88].

The finding of a statistically significant association between therapist adherence and child and adolescent psychotherapy outcomes is in line with the perspective of proponents of ESTs, as this suggests that maintaining a certain level of adherence may be an important component of implementing manualised interventions. On the other hand, the small size of effect is in line with the view by proponents of common factors in psychotherapy that specific therapy model factors contribute relatively little to treatment outcome [89], which is likely to be more strongly associated with a number of factors other than adherence such as client characteristics, e.g. baseline problem severity [90], or therapeutic processes such as therapeutic alliance [91].

There are a number of reasons why caution is needed before drawing conclusions about the relative contribution of model specific and common psychotherapy factors in child outcomes. First and foremost, the relative role of these factors was not the focus of the present study. In addition, most existing studies are not generally designed to test the role of adherence as their primary hypothesis. Further, for most included studies many of the intervention non-specific processes known to be associated with outcome, such as therapeutic alliance [16], were not controlled for and as such may confound any observed relationship between adherence and outcome [80]. In addition to this, common factors such as therapeutic alliance which are seen to be associated with outcome in wider research are also often observed to be small in size [92]. This suggests that common and specific factors may both play small but significant roles in contributing to outcomes. However, while small, mean effects of therapeutic alliance are still often slightly larger than the mean effect size observed in the present study between adherence and outcome.

The small effect seen in the present study may also relate to methodological bias in the included studies which may have led to an under or over-estimation of the relationship between adherence and outcome. In the absence of consistent guidelines there is variation in the methods, reliability and validity of implementation measures varies across studies. For example, adherence was often rated by therapist or clients rather than independent observers and where independent observers were used, inter-rater agreement was not always well established. Studies which measure average implementation or change over time may also provide more accurate evidence than those for which ratings are based on a single session. A sensitivity analysis showed that when excluding studies at highest risk of bias the overall association between adherence and outcome remained a similar size. This indicated that overall study quality was unlikely to be driving the observed effect, although heterogeneity was found to be reduced indicating that quality may contributing at least in part to the variance in effects. Given this, improved reliability and consistency in therapist adherence measures could still further strengthen the reliability of conclusions.

Small effects may also be due to minimal variance in therapist adherence reported in a number of studies, given that therapist adherence was often reported to be moderate to high, thereby reducing power to detect the effect of low adherence. A threshold level may also exist whereby therapist adherence becomes sufficient to achieve optimal outcomes but is no more effective as it increases past this point. For example, the review by Durlak and DuPre [93] suggested that once a sufficient threshold of around 60–80% implementation had been reached, further improvement did not necessarily lead to significantly better outcomes. Furthermore, there may be specific components of adherence in some interventions which are more strongly related to improved outcome than others. For example, in supportive-expressive group counselling, Shechtman and Leichtentritt [45] found that while therapist adherence to intervention components involving encouragement and self-disclosure improved anxiety and social competence, respectively, one component of adherence, challenge, had a negative impact on youth aggression and academic outcome. This would suggest that not all aspects of a given model may be linked to improved youth outcome and a composite measure may be masking effects.

### Limitations

The non-significant moderator effects should be interpreted with caution given the heterogeneous nature of the moderator categories and small number of sample sizes in each. Up to ten effect sizes per moderation category has been indicated as an ideal target [32], which is well above the numbers of studies available for the present analyses. Given the small number of included studies, in order to achieve a reasonable number of studies in each category, the categories of intervention type into which studies were classified were relatively heterogeneous. For example there were insufficient studies to use separate categories for anxiety or depressive disorders, which were instead combined into a broad category of emotional disorder. Use of heterogeneous moderator categories was necessary to allow a moderator analysis to be conducted, but may have limited the validity of the moderator categories used in this analysis. Expansion of the evidence base to increase the number of studies and to enable more precise categorisations would improve the reliability and sensitivity of the test for moderation, allowing improved understanding of the reasons for the observed variation.

This meta-analysis was also unable to test for a number of other aspects of the relationship between therapist implementation and outcome, given the small number of studies in which they were measured. This includes the potential curvilinear relationship between adherence and outcome, such that moderate therapist adherence may be preferable to both very high and very low adherence. Furthermore, given that few studies of child and adolescent psychotherapy have considered the interaction between adherence and therapeutic alliance or competence, the present analysis was not able to consider the role of these moderators in the relationship between adherence and outcome.

Unpublished works such as dissertations were excluded from this meta-analysis on the basis of limited full text availability. Although findings indicated no risk of publication bias, the small sample may affect the reliability of this conclusion, and findings from the sensitivity analysis suggest that correction for publication bias could invalidate the present conclusions. In line with this, Webb et al. [16], who found no overall relationship of therapist adherence and competence with outcome, did include unpublished dissertations. Systematic measurement and reporting of implementation constructs in published research should be encouraged in order that this question can be more rigorously tested. Another limitation in the search approach was restricting the search to English language only, which could have excluded potentially relevant studies. A further limitation of the search approach was that the searches, data extraction and quality ratings were carried out by the lead author only. It was, therefore, not possible to test for inter-rater agreement on these judgements. Finally, conservatively estimating zero for non-significant effects with insufficient data to compute an effect size, to avoid excluding non-significant findings and over-estimating effects, may lead to under estimation of effects if these studies had been under powered to detect associations.

## Conclusion

This study, aiming to understand whether treatment outcomes can be accounted for by therapist adherence to the treatment model or competence in delivery, extends the findings of existing reviews in the field of child and adolescent intervention through inclusion of a broader and more extensive range of studies and use of a more rigorous statistical analysis of findings. The findings indicate that there may be value added in using training and supervision as strategies to ensure the successful transportation and implementation of manualised intervention models across settings. However, the findings also indicate that measures to ensure adherence would only contribute to a small proportion of outcome variance and that there are, therefore, likely to be other intervention non-specific characteristics which may play an equally or more important role than therapist adherence in achieving therapeutic outcomes. These non-specific factors may include client motivation, severity of the presenting problem, the level of therapists’ experience and knowledge of the specific problem or therapeutic alliance. This may suggest the importance of considering both intervention-specific and non-specific factors to achieve outcome in child and adolescent psychotherapy.

Although observed heterogeneity indicates that the adherence–outcome relationship is not universal, this study was unable to identify the moderating factors explaining the observed variation in adherence–outcome effect sizes. As such, future research should aim to further consider the circumstances under which the relationship between adherence and outcome is observed in everyday clinical practice. More consistent reporting of adherence in intervention research, using robust observer-rated adherence measures, would ensure sufficient numbers of valid and reliable effects to enable robust tests for moderation through meta-analysis. Further, factors such as youth risk, therapeutic alliance and competence, which may potentially interact with adherence effects, should be more consistently measured and controlled for to enable an understanding of the adherence–outcome relation over and above the influence of these factors.

Ultimately, what is needed are studies that are specifically designed to evaluate the role of adherence and competence in everyday clinical practice where there is likely to be a greater variability both in the way the treatments are delivered and in factors that may moderate the impact of adherence and competence.

## Electronic supplementary material

Below is the link to the electronic supplementary material.

Supplementary material 1 (pdf 117 kb)

Supplementary material 2 (pdf 230 kb)

Supplementary material 3 (pdf 178 kb)

Supplementary material 4 (pdf 171 kb)
