# Supplementary material for: Systematic literature review and meta-analysis of the relationship between adherence, competence and outcome in psychotherapy for children and adolescents
Source: Eur Child Adolesc Psychiatry. 2019 Jan 2;29(4):417–31. doi: 10.1007/s00787-018-1265-2 (PMC7103576; doi:10.1007/s00787-018-1265-2)
Supplement: Supplementary file 1 — Supplementary material 1 (pdf 117 kb) [file 787_2018_1265_MOESM1_ESM.pdf]

Systematic Literature Review and Meta-Analysis of the Relationship between Adherence, Competence and Outcome in Psychotherapy for Children and Adolescents

European Child and Adolescent Psychiatry

Hannah Collyer[a]

Ivan Eisler[a]

Matt Woolgar[a]

[a]Institute of Psychiatry, Psychology and Neuroscience, King's College London, London, UK

Correspondence concerning this article should be addressed to hannah.collyer@kcl.ac.uk

Appendix 1 : Search Terms

(((Fidelity or adherence or integrity or competence or implementation) and (treatment or therapy or psychotherapy or intervention or therapist or therapeutic or counselling) and (child or adolescent or parent or parenting or teenager or teenage or children or adolescents or youth)) not (HIV or antiretroviral or diabetes or diabetic or cardiac or cardiovascular or vascular or coronary or heart or hypertension or haemophilia or hemophilia or blood or Anemia or Plasma or Pulmonary or Vein or Veins or Artery or Arterial or Ventricular or cholesterol or cancer or tumour or tumor or Chemotherapy or radiation or radiotherapy or asthma or TB or tuberculosis or epilepsy or stroke or sclerosis or hepatitis or cystic or malaria or pneumonia or viral or antiviral or Vaccine or Infection or Infections or Bacteria or Bacterial or Immunological or Immunotherapy or Immunodeficiency or Allergy or Allergic or antibiotic or protein or cell or cells or molecular or Lipid or Acid or Carbon or Cyst or Membrane or rat or mice or mouse or rats or bone or osteoporosis or arthritis or osteoarthritis or spinal or Fracture or Fractures or muscular or skin or liver or kidney or renal or dialysis or Bladder or Urine or Gastric or Ulcer or Ulcers or Gastrointestinal or surgery or surgical or wound or surgeon or surgeons or Anesthetic or Anesthesia or Endoscopic or Endoscopy or Oncology or Radiology or Urology or Donor or Inject or Injection or dental or Dentistry or Tooth or Teeth or Retina or Visual or Eye or Eyes or Glaucoma or Optical or Optic or respiratory or oxygen or lung or reproduction or fertility or contraceptive or vitamin)).ab,kw,sh,ti.
