# Supplementary material for: Systematic literature review and meta-analysis of the relationship between adherence, competence and outcome in psychotherapy for children and adolescents
Source: Eur Child Adolesc Psychiatry. 2019 Jan 2;29(4):417–31. doi: 10.1007/s00787-018-1265-2 (PMC7103576; doi:10.1007/s00787-018-1265-2)
Supplement: Supplementary file 2 — Supplementary material 2 (pdf 230 kb) [file 787_2018_1265_MOESM2_ESM.pdf]

Systematic Literature Review and Meta-Analysis of the Relationship between Adherence, Competence and Outcome in Psychotherapy for Children and Adolescents

European Child and Adolescent Psychiatry

Hannah Collyer[a]

Ivan Eisler[a]

Matt Woolgar[a]

[a]Institute of Psychiatry, Psychology and Neuroscience, King's College London, London, UK

Correspondence concerning this article should be addressed to hannah.collyer@kcl.ac.uk

Appendix 2 : Study Characteristics

Study	Client Group	Intervention	Location	N	Clinical Group Age Range (Mean, *SD*)	RCT	Length of Follow-up	Adherence / Competence		Outcome Measure(s) used in Meta-Analysis
								Informant (inter-rater reliability)	Measure	
Al, Stams, Asscher, and van der Laan (2014)	Families in crisis with concerns about child safety	Family crisis intervention program (FCIP)	Netherlands	183	0-18 (11.54, 5.17)	N	Post intervention	Therapist	Implementation of six components of FCIP	Parent & Youth SDQ
Bloomquist et al. (2013)	Adjustment / aggressive / disruptive / socially withdrawn	Early risers "skills for success" program	US	262	Grade 1 & 2 (6.92, 0.93)	N	1 & 2y post baseline (2y intervention)	Adherence - therapist competence - supervisor (ICC = .62)	Extent & quality of delivery of behavioural change strategies	Teacher Rated BASC-2
Boyer et al. (2018)	Attention-Deficit/Hyperactivity Disorder (ADHD)	CBT (Solution-Focused Treatment)	Netherlands	36	12-17 (14.28, 1.19)	Y	Post intervention	Observer (ICC = .74)	MITI 3.1.1 (competence scales only)	Plan/Organize scale of the Dutch BRIEF
Dagenais, Briere, Gratton, and Dupont (2009)	Behavioural problems in families in crisis	Family brief and intensive intervention (BII)	Canada	160	06-17 (14.7, 2.1)	N	6m follow-up	Therapist	Eight core BII principles (yes / no)	Placement / services required

Study	Client Group	Intervention	Location	N	Clinical Group Age Range (Mean, *SD*)	RCT	Length of Follow-up	Adherence / Competence		Outcome Measure(s) used in Meta-Analysis
								Informant (inter-rater reliability)	Measure	
Eames et al. (2009) Eames et al. (2010)	Prevention & intervention for conduct Disorder	Incredible years parent group	UK	86	36 - 59 mths (46.1mths, 6)[a]	Y	3m follow-up	Observer (inter-rater agreement of ≥ 70% during training)	LOT	Parent Rated ECBI Intensity and Problem Scales
Eisen et al. (2013)	Risk of depressive disorder	Youth single session brief MI	US	43	14-21 (17.44, 2.17)	Y	3m follow-up	Observer (consensus of two raters)	MITI-3 (composite fidelity)	Youth CES-D-10
Garner et al. (2009)	Substance abuse	Youth A-CRA (& parent & family sessions)	US	399	12-18 (N/A)	Y	Post intervention & 3m follow-up	Therapist	AES	Alcohol and other drug use
Garner et al. (2012) Campos-Melady, Smith, Meyers, Godley, and Godley (2017)	Substance abuse	Youth A-CRA (& parent & family sessions)	US	953	N/A (15.8, 1.4)	Block[b]	6 m follow-up	Observer (% Agreement: Competence = 95% Adherence = 100%)	Therapist-level A-CRA competence and patient-level target A-CRA (adherence)	Substance Use Remission Status on the GAIN
Gillespie, Huey, and Cunningham (2017)	Substance using youth offenders	MST	US	40	12-17	Y	4m & 12m post baseline	Observer (ICC = .64)	MST Principle Adherence (MPA) Composite	Arrest Record; Self-Report Alcohol & Cannabis Use; SRDS

Study	Client Group	Intervention	Location	N	Clinical Group Age Range (Mean, *SD*)	RCT	Length of Follow-up	Adherence / Competence		Outcome Measure(s) used in Meta-Analysis
								Informant (inter-rater reliability)	Measure	
Gillham, Hamilton, Freres, Patton, and Gallop (2006)	Elevated depression symptoms	Youth group CBT	US	147	11-12 (N/A)	Y	2–4 wks post intervention & 6, 12, 18, & 24m follow-up	Observer (91% Agreement)	Integrity rating created by PRP's developers	Youth-Report CDI
Ginsburg et al. (2012) Becker, Becker, and Ginsburg (2012)	Anxiety disorder	Individual CBT (& parent sessions)	US	17	07-17 (11.12, 2.75)	Y	Post intervention & 1m follow-up	Observer (Trained to a reliability criterion of Kappa = .80)	TATC	Clinician ADIS-C/P; Parent & Youth SCARED
Graham, Carr, Rooney, Sexton, and Wilson Satterfield (2014)	Behavioural problems	FFT	Ireland	98	N/A (14.05, 1.93)	N	Post intervention	Supervisor	FFT TAM	Parent & Youth SDQ
Hartnett, Carr, and Sexton (2016)	Behavioural problems	FFT	Ireland	42	N/A (14.22, 1.45)	Y	Post intervention & 3m follow-up	Supervisor	FFT TAM	Parent SDQ

Study	Client Group	Intervention	Location	N	Clinical Group Age Range (Mean, *SD*)	RCT	Length of Follow-up	Adherence / Competence		Outcome Measure(s) used in Meta-Analysis
								Informant (inter-rater reliability)	Measure	
Helmond et al. (2012)	Incarcerated youth with antisocial behaviour	Youth group CBT	Netherlands & Belgium	89	N/A (15.54, 1.56)	N	Post intervention	Observer (Kappa = .95)	Observation Checklist Program Integrity EQUIP.	Youth Rated IAP-SFO (Social Skills); SRM-SFO (Moral Value & Moral Judgement); HIT (Cognitive Distortion)
Henggeler et al. (1997)[c]	Violent and chronic youth offenders	MST	US	82	10-17 (15.22, N/A) [a]	Y	Post intervention (Offending stats 1.7y follow-up)	Client & therapist composite	MST TAM	Arrests & incarceration; SRDS; Parent RPBC
Henggeler, Pickrel, and Brondino (1999)	Substance using youth offenders	MST	US	58	12-17 (15.7, 1.0) [a]	Y	Post intervention & 6m follow-up	Client & therapist composite	MST TAM	Self-report alcohol / marijuana
Heywood and Fergusson (2016)	Behavioural Problems	FFT	New Zealand	59	9-16 (13y7m)	N	6m & 12m post baseline	Supervisor	Global Therapist Rating Scale (GTRS)	Combined measure of parent rated conduct problems,

Study	Client Group	Intervention	Location	N	Clinical Group Age Range (Mean, *SD*)	RCT	Length of Follow-up	Adherence / Competence: Informant (inter-rater reliability)	Adherence / Competence: Measure	Outcome Measure(s) used in Meta-Analysis
										parent & youth rated alcohol & substance use frequency, and SRDS
Hogue et al. (2008)[d]	Substance use and behaviour problems	Family MDFT & youth CBT	US	74 MDFT, 62 CBT	13-17 (15.5, 1.3)	Y	Post intervention & 6m & 12m follow-up	Observer ICC Adherence CBT = 56-.83 MDFT = .64-.79 Competence CBT = .01-.63 MDFT = .15-.48	TBRS – C	TLFB cannabis Frequency
Holth, Torsheim, Sheidow, Ogden, and Henggeler (2011)	Substance use disorder	MST	Norway	41	13-17 (15y10m, 14m)	Block	During & post intervention	Client & therapist composite	CM/CBT-TAM	Self-report cannabis abstinence

Study	Client Group	Intervention	Location	N	Clinical Group Age Range (Mean, *SD*)	RCT	Length of Follow-up	Adherence / Competence		Outcome Measure(s) used in Meta-Analysis
								Informant (inter-rater reliability)	Measure	
Hukkelberg and Ogden (2013) Forgatch and DeGarmo (2011)	Externalising behaviour	Parent management training (individual)	Norway	331	04-12 (8.7, N/A)	N	Post intervention	Observer (Kappa = .67)	FIMP	Parent CBCL & PDR; Teacher TRF
Lange et al. (2017) Lange et al. (2018)	Antisocial or delinquent problem behaviour	MST	Netherlands	4,290	12-17 (15.62, 1.38)	N	End of therapy	Caregiver	TAM	Living at home, Engaged in school or work, No new arrests
Liber et al. (2010)	Anxiety disorder	Individual & group CBT (& parent sessions)	Netherlands	52	08-12 (10.22, 1.15)	Y	1m follow-up	Observer (Yule's Y = .68)	Dutch translated FRIENDS treatment adherence protocol	Clinician ADIS-C/P; Youth MASC; Parent CBCL Internalising Scale
Lofholm et al. (2014) Sundell et al. (2008)	Severe behaviour problems or conduct disorder (inc. juvenile offenders)	MST	Sweden	973	12-17 (N/A)	N	Post intervention	Caregiver	MST TAM & TAM-R	Criminal behaviour at completion of treatment
Maaskant, van Rooij, Overbeek, Oort,	Foster children with severe externalising behaviour	Parent Management Training	Netherlands	46	4-12 (7.85, 2.36)	Y	Post intervention & 4m follow-up	Supervisor	FIMP	Parent rated CBCL & teacher rated

Study	Client Group	Intervention	Location	N	Clinical Group Age Range (Mean, *SD*)	RCT	Length of Follow-up	Adherence / Competence		Outcome Measure(s) used in Meta-Analysis
								Informant (inter-rater reliability)	Measure	
and Hermanns (2016)		Oregon (PMTO)								TRF (Dutch versions)
McCambridge et al. (2011)	Cannabis use	Youth single session MI	UK	75	16-19 (18, N/A)	Y	3m follow-up	Observer Adherence ICC = .56-.98 Competence ICC = .74 - .91	MITI-2 (separate competence & adherence)	Cannabis cessation
Overbeek, de Schipper, Lamers-Winkelman, and Schuengel (2013)	Exposure to interparental violence	Youth & parent trauma-focused psycho-education	Netherlands	100	06-12 (9.35, 1.55)	Y	Post intervention & 6m Follow-Up	Observer (insufficient inter-rater data)	Rated on deviations from the manual	Parent Report TSCYC; Youth Report TSCC
Podell et al. (2013)	Anxiety disorder	Youth individual CBT (& parent sessions)	US	279	07-17 (10.76, 2.79)	Y	Post intervention	Observer (insufficient inter-rater data)	CBTC	Clinician PARS; Youth MASC; Parent CBCL Anxiety/Depression Scale
Robbins et al. (2011)	Drug abuse	BSFT	US	246	12-17 (15.5, 1.3)	Y	Up to 12m post baseline	Observer (ICC = .83)	BSFT TAF	TLFB Substance Use

Study	Client Group	Intervention	Location	N	Clinical Group Age Range (Mean, *SD*)	RCT	Length of Follow-up	Adherence / Competence		Outcome Measure(s) used in Meta-Analysis
								Informant (inter-rater reliability)	Measure	
Rowe et al. (2013)	Cannabis use disorder	MDFT	Europe & US	Europe (212) US (171)	13-18 (Europe: 16.3, 1.2) [a] (US: 15.6, 1.1)	Y	12m follow-up	Observer (ICC = .81)	MDFT Intervention Inventory	TLFB Substance Use Frequency & Problem
Schoenwald, Sheidow, et al. (2003)[e]	Serious antisocial behaviour	MST	US	1979	N/A (14.0, 2.35)	N	Post intervention 6m, 1y, 4y follow-up	Caregiver	MST TAM & TAM-R	Parent CBCL Externalising
Sexton and Turner (2010)	Youth offenders	FFT	US	431	13-17 (N/A)	Y	6-9m follow-up	Supervisor	FFT TAM	Recidivism
Shechtman and Leichtentritt (2010)	Social, emotional, and behavioural problems	Youth supportive-expressive group counselling	Israel	266	10-18 (N/A)	N	Post intervention	Youth	Adapted CHSM	Self-report aggression
Smith, Dishion, Shaw, and Wilson (2013) Chiapa et al. (2015)	Elevated problem behaviour	Family check-up	US	79	2y0m-2y11m (29.9 mths, 3.2)	Y	Fidelity at age 3,4 & 5 & follow-up at age 7.5 & 8.5	Observer (ICC = .74)	COACH observational rating system	Parent CBCL Externalising

Study	Client Group	Intervention	Location	N	Clinical Group Age Range (Mean, *SD*)	RCT	Length of Follow-up	Adherence / Competence		Outcome Measure(s) used in Meta-Analysis
								Informant (inter-rater reliability)	Measure	
Strauss et al. (2012) Strauss et al. (2015)	Autism spectrum disorder	Youth & parent EIBI	Italy	24	26-81 months (55.67 months, 17.63)	N	6m, 1y post baseline	Observer (ICC = .89 - .96)	Adherence checklist; OPTION competence measure	Observer rated child challenging behaviour
Williams and Green (2012)	Serious emotional disturbance	Youth & parent CPSR	US	79	N/A (10.31, 3.54)	N	3wk follow-up	Parent	CTAM	Parent Rated YCIS

[a] Age is reported for whole sample including control

[b] Block randomisation (by site)

[c] Study also reported in Schoenwald, Henggeler, Brondino, and Rowland (2000), Huey, Henggeler, Brondino, and Pickrel (2000)

[d] Study also reported in Hogue, Liddle, Dauber, and Samuolis (2004), Hogue et al. (2006)

[e] Study also reported in Schoenwald, Carter, Chapman, and Sheidow (2008), Schoenwald, Chapman, Sheidow, and Carter (2009), Schoenwald, Sheidow, and Chapman (2009), Halliday-Boykins et al. (2005), Chapman and Schoenwald (2011)

Interventions: A-CRA = Adolescent Community Reinforcement Approach; BSFT = Brief Strategic Family Therapy; CBT = Cognitive Behavioural Therapy; CPSR = Child psychiatric rehabilitation; EIBI = Early Intensive Behavioural Intervention; FFT = Functional Family Therapy; MDFT = Multidimensional Family Therapy; MI = Motivational Interviewing; MST = Multisystemic Therapy;

Adherence / Competence Measures: AES = A-CRA Exposure Scale; CBTC = CBT Checklist; CHSM = Counsellor Helping Skills Measure; CTAM = Children's Psychosocial Rehabilitation Treatment Adherence Measure; FIMP = Fidelity of Implementation Rating Scale; LOT = Leader Observation Tool; MITI-2/3 = Motivational Interview Treatment Integrity 2/3; OPTION = Observing Patient Involvement Scale; TAF = Therapist Adherence Form; TAM(-R) = Therapist Adherence Measure (Revised); TATC = Treatment Adherence and Therapist Competence; TBRS-C = Therapist Behavior Rating Scale—Competence;

Outcome Measures: ADIS-C/P = Rated Anxiety Disorders Interview Schedule for DSM-IV, Parent and Child Versions; BASC-2 = Behavioural Assessment System for Children-II; BRIEF = Behaviour Rating Inventory of Executive Function; CBCL = Child Behaviour Checklist; CDI – Clinical Depression Inventory; CES-D-10 = 10-item Center for Epidemiologic Studies Depression Scale; ECBI = Eyberg Child Behaviour Inventory; GAIN = Global Appraisal of Individual Needs; HIT = How I Think Questionnaire; IAP-SFO = Inventory of Adolescent Problems — Short Form Objective; MASC = Multidimensional Anxiety Scale for Children; PARS = Pediatric Anxiety Rating Scale; PDR = Parent Daily Report; RPBC = Revised Problem Behaviour Checklist; SCARED = Screen for Child Anxiety—Related Emotional Disorders; SDQ = Strength & Difficulties Questionnaire; SRDS = Self Report Delinquency Scale; SRM-SFO = Sociomoral Reflection Measure — Short Form Objective; TLFB = Time Line Follow-Back; TRF = Teacher Report Form; TSCC = Trauma Symptom Checklist for Children; TSCYC = Trauma Symptom Checklist for Young Children; YCIS = Youth Counseling Impact Scale;
