# Supplementary material for: Systematic literature review and meta-analysis of the relationship between adherence, competence and outcome in psychotherapy for children and adolescents
Source: Eur Child Adolesc Psychiatry. 2019 Jan 2;29(4):417–31. doi: 10.1007/s00787-018-1265-2 (PMC7103576; doi:10.1007/s00787-018-1265-2)
Supplement: Supplementary file 3 — Supplementary material 3 (pdf 178 kb) [file 787_2018_1265_MOESM3_ESM.pdf]

Systematic Literature Review and Meta-Analysis of the Relationship between Adherence, Competence and Outcome in Psychotherapy for Children and Adolescents

European Child and Adolescent Psychiatry

Hannah Collyer[a]

Ivan Eisler[a]

Matt Woolgar[a]

[a]Institute of Psychiatry, Psychology and Neuroscience, King's College London, London, UK

Correspondence concerning this article should be addressed to hannah.collyer@kcl.ac.uk

Appendix 3 : Intervention Details

Study	Client Group Moderator Classification	Intervention	Moderator Classification	Description
Al et al. (2014)	Child or adolescent behaviour	Family crisis intervention program (FCIP)	Family Therapy	A brief, intensive in-home intervention focusing on the whole family in line with the system approach and network approach.
Boyer et al. (2018)	Child or adolescent behaviour	Cognitive Behavioural Therapy (Solution-Focused Treatment)	CBT	10 sessions (n = 8 adolescent; n = 2 parent), 45 minutes long. The first session involved psychoeducation and formulating treatment goals. Each session discussed a problem encountered in the past week, using fixed questions to guide toward a solution. Motivational Interviewing (MI) was integrated into the treatment to reduce dropout.
Bloomquist et al. (2013)	Child or adolescent behaviour	Early risers "skills for success" program	Parent & Youth Intervention (non-CBT)	A coordinated package of child-focused skills training and school support interventions, along with group based parent education/skills training and family support interventions over a two-year period. Integrates social learning, social development, cognitive behavioural, developmental, and parenting models.
Dagenais et al. (2009)	Child or adolescent behaviour	Family brief and intensive intervention (BII)	Family Therapy	Family-centred interventions (multiple family members involved in program activities) using an eclectic range of strategies and techniques including active listening, cognitive restructuring, paradox, and conflict management techniques.
Eames et al. (2009)	Child or adolescent behaviour	Incredible years parent group	Parenting	12-week parenting group intervention. 2 leaders per group. Groups of up to 12. Core components are play and relationship building, praise and reward, effective limit setting and handling misbehaviour.

Study	Client Group Moderator Classification	Intervention	Moderator Classification	Description
Eisen et al. (2013)	Emotional disorder	Youth single session brief MI	Youth Intervention (non-CBT)	Single session motivational interviewing: 6 to 15-minute intervention which used a nondirective approach, to help the adolescent develop a favourable cost/benefit assessment toward completing the internet based intervention and build resiliency.
Garner et al. (2009)	Substance use	Youth A-CRA (& parent & family sessions)	Parent & Youth Intervention (non-CBT)	An adaptation of the adult community reinforcement approach, a behavioural therapy, incorporating youth, parent, and family sessions.
Garner et al. (2012)	Substance use	Youth A-CRA (& parent & family sessions)	Parent & Youth Intervention (non-CBT)	An adaptation of the adult community reinforcement approach, a behavioural therapy, incorporating youth, parent, and family sessions.
Gillespie et al. (2017)	Substance use	MST	Family Therapy	An intensive family and community based intervention
Gillham et al. (2006)	Emotional disorder	Youth group CBT	CBT	Youth group therapy largely based on cognitive-behavioural theories.
Ginsburg et al. (2012)	Emotional disorder	Individual CBT (& parent sessions)	CBT	Child cognitive behavioural therapy based on 'Coping Cat'. Mainly child but some parent sessions
Graham et al. (2014)	Child or adolescent behaviour	FFT	Family Therapy	Family therapy intervention for the treatment of violent, criminal, behavioural, school, and conduct problems with youth and their families.
Hartnett et al. (2016)	Child or adolescent behaviour	FFT	Family Therapy	Family therapy intervention for the treatment of violent, criminal, behavioural, school, and conduct problems with youth and their families.

Study	Client Group Moderator Classification	Intervention	Moderator Classification	Description
Helmond et al. (2012)	Child or adolescent behaviour	Youth group CBT	CBT	A cognitive behavioural youth intervention designed to target cognitive distortions, social skill deficiencies and moral developmental delays.
Henggeler et al. (1997)	Child or adolescent behaviour	MST	Family Therapy	An intensive family and community based intervention
Henggeler et al. (1999)	Substance use	MST	Family Therapy	An intensive family and community based intervention
Heywood and Fergusson (2016)	Child or adolescent behaviour	FFT	Family Therapy	Family therapy intervention for the treatment of violent, criminal, behavioural, school, and conduct problems with youth and their families.
Hogue et al. (2008)	Substance use	Family MDFT	Family Therapy	A developmentally oriented outpatient treatment which uses a combination of family therapy, individual therapy, drug counselling and multiple-systems oriented interventions
		Youth CBT	CBT	Based on a broadly defined cognitive-behavioural framework and was also influenced by dialectical behaviour therapy.
Holth et al. (2011)	Substance use	MST	Family Therapy	An intensive family and community based intervention
Hukkelberg and Ogden (2013)	Child or adolescent behaviour	Parent management training (individual)	Parenting	A parenting intervention based on social interaction learning theory with components addressing positive and negative contingencies for behaviour, emotional regulation, problem-solving and academic achievement.

Study	Client Group Moderator Classification	Intervention	Moderator Classification	Description
Lange et al. (2017)	Child or adolescent behaviour	MST	Family Therapy	An intensive family and community based intervention
Liber et al. (2010)	Emotional disorder	Individual & group CBT (& parent sessions)	CBT	A Dutch translation of FRIENDS programme, based upon the cognitive behavioural 'Coping Cat' program. Individual and group youth and parent sessions.
Lofholm et al. (2014)	Child or adolescent behaviour	MST	Family Therapy	An intensive family and community based intervention
Maaskant et al. (2016)	Child or adolescent behaviour	Parent Management Training Oregon (PMTO)	Parenting	An intensive and individualised parent training based on the social interaction learning model. The main focus is enhancing effective and positive parenting practice and diminishing coercive practices. In this study the intervention was implemented with foster carers.
McCambridge et al. (2011)	Substance use	Youth single session MI	Youth Intervention (non-CBT)	A motivational interviewing early intervention model adapted for preventative purposes
Overbeek et al. (2013)	Emotional disorder	Youth & parent trauma-focused psycho-education	Parent & Youth Intervention (non-CBT)	Parent group sessions focussed on psycho-education and discussion as well as a focus on improving parenting quality and reducing children's adjustment problems. Child group sessions focussed on processing violence experiences, learning how to differentiate and express emotions and learning to cope with feelings and problems in a nonviolent way.
Podell et al. (2013)	Emotional disorder	Youth individual CBT (& parent sessions)	CBT	Cognitive behavioural 'Coping Cat' program / 'C.A.T. Project' for teens. Mainly child but some parent sessions.

Study	Client Group Moderator Classification	Intervention	Moderator Classification	Description
Robbins et al. (2011)	Substance use	BSFT	Family Therapy	A problem focussed intervention which combines structural and strategic family therapy theory and intervention techniques with the aim of improving family functioning and relationships between family and important external systems (such as school, peers).
Rowe et al. (2013)	Substance use	MDFT	Family Therapy	A developmentally oriented outpatient treatment which uses a combination of family therapy, individual therapy, drug counselling and multiple-systems oriented interventions
Schoenwald, Sheidow, et al. (2003)	Child or adolescent behaviour	MST	Family Therapy	An intensive family and community based intervention
Sexton and Turner (2010)	Child or adolescent behaviour	FFT	Family Therapy	Family therapy intervention for the treatment of violent, criminal, behavioural, school, and conduct problems with youth and their families.
Shechtman and Leichtentritt (2010)	Child or adolescent behaviour	Youth supportive-expressive group counselling	Youth Intervention (non-CBT)	A group intervention adjusted for young clients from an eclectic supportive-expressive modality incorporating client-centred, psychodynamic, and cognitive behavioural approaches.
Smith et al. (2013)	Child or adolescent behaviour	Family check-up	Parent	Brief (3 session) ecological intervention using parent management training in use of positive behaviour support.

Study	Client Group Moderator Classification	Intervention	Moderator Classification	Description
Strauss et al. (2012)	Autism spectrum disorder	Youth & parent EIBI	Parent & Youth Intervention (non-CBT)	25 h/week in centre-based one-to-one and play room setting and at least 10 h/week in home-based setting in a one week centre / three weeks home rhythm for 12 months. Comprises the systematic use of discrete trail teaching and more natural approaches such as incidental teaching, and natural environment teaching.
Williams and Green (2012)	Emotional disorder	Youth & parent CPSR	Parent & Youth Intervention (non-CBT)	A home- and community-based skill-training program also working with caregivers and other important adults in the child's life to modify the child's environment to support the use of adaptive skills and behaviours. Focus on skills training (cognitive and behavioural) and motivation enhancement for the youth their caregiver(s).
