# Supplementary material for: Systematic literature review and meta-analysis of the relationship between adherence, competence and outcome in psychotherapy for children and adolescents
Source: Eur Child Adolesc Psychiatry. 2019 Jan 2;29(4):417–31. doi: 10.1007/s00787-018-1265-2 (PMC7103576; doi:10.1007/s00787-018-1265-2)
Supplement: Supplementary file 4 — Supplementary material 4 (pdf 171 kb) [file 787_2018_1265_MOESM4_ESM.pdf]

Systematic Literature Review and Meta-Analysis of the Relationship between Adherence, Competence and Outcome in Psychotherapy for Children and Adolescents

European Child and Adolescent Psychiatry

Hannah Collyer[a]

Ivan Eisler[a]

Matt Woolgar[a]

[a]Institute of Psychiatry, Psychology and Neuroscience, King's College London, London, UK

Correspondence concerning this article should be addressed to hannah.collyer@kcl.ac.uk

Appendix 4 : Study Quality

Study	Power	Risk of Bias in Included Participants			Risk of Bias in Reliability and Validity of Measures		Risk of Bias in Control for Confounding Variables		Number of Items with Lower Quality
	Sample Size	Participation Rate	Attrition or Missing Data	Selection Criteria	Reliability of Implementation Measure	Reliability of Outcome Measure	Adjusted or Controlled for Baseline Severity	Adjusted or Controlled for Other Confounders	
Al et al. (2014)	+	-	-	-	-	+	+	-	5
Bloomquist et al. (2013)	+	+	+	+	-	+	+	-	2
Boyer et al. (2008)	-	-	+	-	+	+	+	+	3
Dagenais et al. (2009)	+	+	+	-	-	-	-	-	5
Eames et al. (2009)	+	+	+	+	-	+	+	-	2
Eisen et al. (2013)	-	-	-	+	+	+	+	+	3
Garner et al. (2009)	+	+	+	+	-	+	+	+	1
Garner et al. (2012)	+	-	-	-	+	+	-	-	5
Gillespie et al. (2017)	-	+	+	+	+	+	+	+	1
Gillham et al. (2006)	+	+	-	+	+	+	+	+	1
Ginsburg et al. (2012)	-	+	+	+	+	+	+	-	2
Graham et al. (2014)	+	-	+	-	-	+	+	+	3
Hartnett et al. (2016)	-	-	-	+	-	+	-	-	6
Helmond et al. (2012)	+	-	-	-	+	-	+	+	4
Henggeler et al. (1997)	+	+	+	+	-	+	+	-	2
Henggeler et al. (1999)	-	+	+	+	-	+	+	-	3
Heywood and Fergusson (2016)	-	+	-	-	-	+	+	-	5
Hogue et al. (2008)	+	+	-	+	-	+	+	+	2
Holth et al. (2011)	-	+	-	+	-	+	+	+	3
Hukkelberg and Ogden (2013)	+	-	-	-	+	+	+	-	4
Lange et al. (2017)	+	+	+	-	-	+	-	+	3

Study	Power	Risk of Bias in Included Participants			Risk of Bias in Reliability and Validity of Measures		Risk of Bias in Control for Confounding Variables		Number of Items with Lower Quality
	Sample Size	Participation Rate	Attrition or Missing Data	Selection Criteria	Reliability of Implementation Measure	Reliability of Outcome Measure	Adjusted or Controlled for Baseline Severity	Adjusted or Controlled for Other Confounders	
Liber et al. (2010)	-	+	+	+	+	+	+	+	1
Lofholm et al. (2014)	+	+	-	-	-	-	-	+	5
Maaskant et al. (2016)	-	-	-	+	-	+	+	-	5
McCambridge et al. (2011)	+	-	+	-	-	+	-	+	4
Overbeek et al. (2013)	+	+	+	-	-	+	+	+	2
Podell et al. (2013)	+	+	+	+	-	+	+	+	1
Robbins et al. (2011)	+	-	-	+	+	+	+	-	3
Rowe et al. (2013)	+	-	+	+	-	+	+	-	3
Schoenwald, Sheidow, et al. (2003)	+	+	+	-	-	+	+	+	2
Sexton and Turner (2010)	+	-	+	-	-	+	+	+	3
Shechtman and Leichtentritt (2010)	+	+	+	-	-	+	+	+	2
Smith et al. (2013)	+	+	-	+	+	+	+	-	2
Strauss et al. (2012)	-	-	+	+	+	-	+	+	3
Williams and Green (2012)	-	-	+	-	-	+	+	+	4
N of studies at risk (of 35)	12 (34%)	15 (43%)	14 (40%)	16 (46%)	23 (66%)	4 (11%)	6 (17%)	15 (43%)	

‘+‘ = lower risk of bias or higher quality ; ‘-’ = higher risk of bias or lower quality (see methods section for criteria)
